# The Relationship Between Environmental Exposure and Genetic Architecture of the 2q33 Locus With Esophageal Cancer in South Africa

**DOI:** 10.3389/fgene.2019.00406

**Published:** 2019-05-01

**Authors:** Marco Matejcic, Christopher G. Mathew, M. Iqbal Parker

**Affiliations:** ^1^Division of Medical Biochemistry and Structural Biology, Institute for Infectious Disease and Molecular Medicine, University of Cape Town, Cape Town, South Africa; ^2^Sydney Brenner Institute for Molecular Bioscience, University of the Witwatersrand, Johannesburg, South Africa; ^3^Department of Medical and Molecular Genetics, Faculty of Life Sciences and Medicine, King’s College London, London, United Kingdom

**Keywords:** genetic association, single nucleotide polymorphism, esophageal squamous cell carcinoma, South African populations, major histocompatibility complex, 2q33, TRAK2

## Abstract

Esophageal squamous cell carcinoma (ESCC) has a high prevalence in several countries in Africa and Asia. Previous genome-wide association studies (GWAS) in Chinese populations have identified several ESCC susceptibility loci, including variants on chromosome 2q33 and 6p21, but the contribution of these loci to risk in African populations is unknown. In this study we tested the association of 10 genetic variants at these two risk loci on susceptibility to ESCC in two South African ethnic groups. Variants at 2q33 (rs3769823, rs10931936, rs13016963, rs7578456, rs2244438) and 6p21 (rs911178, rs3763338, rs2844695, rs17533090, rs1536501) were genotyped in a set of Black Xhosa (463 cases and 480 controls) and Mixed Ancestry (269 cases and 288 controls) individuals. Genotyping was performed using TaqMan allelic discrimination assays. The Pearson’s chi-squared test was used to compare the allele frequency between cases and controls. Gene-environment interactions with tobacco smoking and alcohol consumption were investigated in a case-control analysis. A logistic regression analysis was further performed to elucidate the independent effect of each association signal on the risk of ESCC. The 2q33 variants rs10931936, rs7578456, and rs2244438 were marginally associated with higher risk of ESCC in the Mixed Ancestry population (ORs = 1.39–1.58, *p* ≤ 0.035), of which rs7578456 and rs2244438 remained significant after multiple correction (*p* < 0.005). The associations with rs7578456 and rs2244438 were also observed across strata of tobacco smoking (ORs = 1.47–2.75, *p* ≤ 0.035) and alcohol consumption (ORs = 1.45–2.06, *p* ≤ 0.085) status. However, only the association with rs2244438, which lies within an exon of *TRAK2*, remained significant after adjustment for the other variants in the region. Interestingly, none of the variants tested were significantly associated with ESCC in the Black South African population. These finding implicate *TRAK2* as a casual gene for ESCC risk in the Mixed Ancestry population of South Africa and confirm prior evidence of population-specific differences in the genetic contribution to ESCC, which may reflect differences in genetic architecture and environmental exposure across ethnic groups.

## Introduction

Esophageal squamous cell carcinoma (ESCC) is one of the most common malignancies in low- and middle-income countries and is a disease of major public health importance because of its poor prognosis and high mortality. The striking variation in the prevalence of ESCC between different ethnic groups is suggestive of contribution by population-specific environmental and dietary factors to susceptibility to the disease. However, although individuals within a specific geographical area may be exposed to the same environmental factors and share similar dietary habits, not all of them have the same risk of developing ESCC. It is clear that a combination of genetic susceptibility and environmental risk factors/diet are key components in the risk of development ESCC (Dandara et al., 2006;Vogelsang et al., 2012, 2014).

Esophageal squamous cell carcinoma (ESCC) accounts for about 90% of the 456,000 esophageal cancer cases reported each year (Abnet et al., 2018), and approximately 80% of the cases worldwide occur in low-to-middle income countries (LMIC) including South Africa (Ferlay et al., 2015; Wong et al., 2018). Tobacco smoking and alcohol consumption are the major environmental risk factors in South Africa (Dandara et al., 2015).

There is also strong evidence for the role of genetic factors in the etiology of ESCC (Sampson et al., 2015). Studies investigating the association between several single nucleotide polymorphisms in several drug metabolizing genes and the risk of developing ESCC have shown a clear population and ethnic variation in the risk profile. More than 200 xenobiotic-metabolizing enzymes are responsible for the metabolism and detoxification of dietary and environmental carcinogens, which if not removed, can bind to DNA and may lead to cancer causing mutations. Genes involved in the biosynthesis of these enzymes all comprise genetic polymorphic variants with altered gene expression or enzyme activity and may serve as molecular biomarkers that can provide important predictive information about carcinogenesis (reviewed in Matejcic and Iqbal Parker, 2015).

The development of genome-wide association studies (GWAS) has had a major impact in the discovery of multiple susceptibility loci for ESCC in Asian and Caucasian populations, including variants in *PLCE1*, *C20orf54*, *PDE4D*, *RUNX1*, and *CASP8* (Abnet et al., 2010; Wang et al., 2010; Wu et al., 2011, 2012). However, the majority of these associations were not found in the Black and Mixed Ancestry populations of South Africa (Bye et al., 2011, 2012; Chen et al., 2019), suggesting the existence of genetic heterogeneity in the risk to ESCC.

A recent GWAS in a northern Chinese population identified common genetic variants on chromosome 2q33 that increased the risk for both ESCC and lung cancer (Zhao et al., 2017). These variants are therefore strong candidates for the study of pivotal biological mechanisms and pleiotropic effects associated with carcinogenesis. Previous GWAS and case-control studies have also identified the human major histocompatibility complex (MHC) region on chromosome 6p21 as a novel susceptibility locus for ESCC in high-risk populations from northern China (Wu et al., 2011; Shen et al., 2014; Zhang et al., 2017). Of these, the variant rs10484761 located upstream of the *UNC5CL* gene was found to be significantly associated with increased risk of ESCC in the Mixed Ancestry South African population (Bye et al., 2012). Nonetheless, the contribution of other risk variants in this region to susceptibility to ESCC in South African population is unknown.

In this study we investigated whether single nucleotide polymorphisms (SNPs) at 2q33 and 6p21 reported to be associated with ESCC in the northern Chinese populations also contributed to the increased risk of ESCC in the South African populations. We explored the interaction between these genetic factors and environmental exposure and used haplotype analyses to investigate the combined effect of these variants on the risk to ESCC. Finally, SNPs with suggestive evidence for association were investigated in a logistic regression model to assess the independent effect of each variant on the risk. TRAK2 was identified as a casual gene for ESCC risk in the Mixed Ancestry population of South Africa, but not in the Black population of Bantu decent, and confirms prior evidence of population-specific differences in the genetic contribution to ESCC.

## Materials and Methods

### Study Group

The study comprised 463 ESCC patients and 480 controls from the Black population and 269 ESCC patients and 288 controls from the Mixed Ancestry population who provided blood samples at recruitment. Black subjects were mainly Xhosa speakers for the last two generations (98.6%) from the Western Cape province of South Africa who migrated from the Eastern Cape over the past 1–2 generations where the majority of Xhosa speakers reside The Mixed Ancestry subjects were from the Western Cape. This is an admixed population that originated ∼300 years ago from the union of different ethnic groups with major ancestral components from the indigenous Khoisan, Bantu-speaking Africans, Europeans and Asians. Analysis of 75,000 autosomal SNPs in the Mixed Ancestry population of the Western Cape (formerly described as the Cape Colored population) compared with populations represented in the International HapMap Project and the Human Genome Diversity Project revealed that the major ancestral components of this population are predominantly Khoisan (32–43%), Bantu-speaking Africans (20–36%), European (21–28%), and a smaller Asian contribution (9–11%). Asian component is mainly from Indonesia, Malaysia and the Indian subcontinent (de Wit et al., 2010). All patients were histologically diagnosed with primary invasive ESCC and were recruited between 2000 and 2012 at Groote Schuur and Tygerberg Hospitals in Cape Town. The control group included healthy volunteers with no history of cancer, and matched to cases for residential area, socioeconomic status, race, sex, and age.

All study participants completed a standardized questionnaire to collect demographic and lifestyle information. Data on alcohol consumption and tobacco smoking were available for both cases and controls. Alcohol drinkers and ever-smokers were defined as subjects who consumed any alcoholic beverage at least once every week, and those who had smoked at some point in their life; otherwise, they were defined as non-drinkers and never-smokers. Demographic and exposure data are presented in Table 1.

**Table 1:** Descriptive characteristics of Black and Mixed Ancestry ESCC cases and controls.

	Black		Mixed Ancestry	
	Cases	Controls	Cases	Controls
Total	463	480	269	288
Mean age (±SD)^a^	59.6 (±10.7)	56.7 (±15.0)	60.7 (±10.3)	57.7 (±14.3)
Sex, n (%)				
Male	229 (49)	235 (49)	177 (66)	178 (62)
Female	234 (51)	245 (51)	92 (34)	110 (38)
Tobacco Smoking, n (%)				
Ever-smokers^b^	280 (61)	222 (46)	250 (94)	226 (78)
Never-smokers	181 (39)	258 (54)	15 (6)	62 (22)
NA^c^	2 (0)	0 (0)	4 (0)	0 (0)
Alcohol Consumption, n (%)				
Drinkers^d^	286 (62)	278 (58)	215 (81)	172 (60)
Non-drinkers	175 (38)	201 (42)	51 (19)	115 (40)
NA^c^	2 (0)	1 (0)	3 (0)	1 (0)

*^a^Age of diagnosis for cases; age of recruitment for controls. ^b^Ever-smokers include subjects who had smoked at some point in their lives. ^c^Not available due to missing data. ^d^Drinkers include subjects who consumed alcohol at least once a week.*

Ethical approval for the study was obtained from the joint University of Cape Town/Groote Schuur Hospital Research Ethics Committee and the University of Stellenbosch/Tygerberg Hospital Ethics Committee. All subjects gave written informed consent in accordance with the Declaration of Helsinki.

### Isolation and Purification of DNA

Peripheral blood samples were collected, with informed consent from all the participants and DNA was extracted at the University of Cape Town using a standard protocol (Gustafson et al., 1987). All DNA samples were diluted to a final concentration of 20 ng/μl in 96-well plates and stored at −20°C until use.

### SNP Selection and Genotyping

For the 2q33 locus, we selected five SNPs (rs3769823, rs10931936, rs13016963, rs7578456, and rs2244438) that were significantly associated (p < 0.05) with both esophageal and lung cancer risk in a northern Chinese population (Zhao et al., 2017). For the 6p21 locus, the selected SNPs were those with strongest evidence of association from published studies of ESCC in northern China (Shen et al., 2014; Zhang et al., 2017). These risk variants were investigated for their frequency in sample populations from the 1000 Genomes Project Phase 3 ^1^. We removed one SNP at 6p21 (rs6901869) that failed the frequency test (<5% frequency in African populations) as we would have limited power to detect any association of this SNP with ESCC. Since rs17533090 at 6p21 was highly correlated with rs35399661 in both Chinese and African populations (D’ = 1/*r*^2^ = 1 in 1000 Genome Project data), only rs17533090 was genotyped as a proxy for rs35399661. Finally, five SNPs from the 6p21 locus (rs17533090, rs911178, rs2844695, rs1536501, and rs3763338) were selected for genotyping.

Genotyping was performed using validated TaqMan allele discrimination assays (Applied Biosystems). Reactions were carried out in 2.5 μl volumes in 96-well plates. Each reaction contained 20 ng DNA diluted in distilled water (dH_2_O), 1X Universal PCR Master Mix, and 1X TaqMan SNP assay mix containing primers and TaqMan probes according to the manufacturer’s protocol. The thermal cycling conditions consisted of an initial denaturation step at 95°C for 10 min followed by 40 cycles of a two-step reaction: denaturation at 92°C for 15 s and annealing/extension at 60°C for 60 s. Amplification reactions and fluorescence measurements at the PCR end-point were performed in a Roche LightCycler 480 II instrument, and genotypes assigned using SP4 1.5.0 software (Roche). Genotype distributions in controls were tested for deviation from Hardy-Weinberg Equilibrium (HWE) using the Pearson’s chi-squared test with a cut-off of *p* < 0.001. All genotype frequencies were in HWE in both ethnic groups. Call rates for all SNPs genotyped were > 95%.

### Statistical Analysis

Allele frequencies in cases and controls by ancestry group were compared using the Pearson’s chi-squared test for association with ESCC. Odds ratios (OR) and 95% confidence intervals (CI) were estimated using the common allele as the reference. A Bonferroni-corrected *P*-value of < 0.005 (0.05/10) was used to determine the significance threshold for all association tests based on the number of SNPs tested. For SNPs with marginal association (*p* < 0.05), gene-environment interactions were investigated in a case-control analysis stratified by tobacco smoking and alcohol consumption status. Haplotypes and correlation coefficients (D^*I*^ and *r*^2^) in controls were estimated using Haploview 4.2 (Barrett et al., 2005). The haplotype analysis was performed using UNPHASED (Dudbridge, 2008). Only haplotypes with an estimated frequency in controls ≥5% were tested. A logistic regression analysis was then performed to examine the independence of association evidence using the cancer status as a binary, dependent variable (affected or unaffected) and the SNP as independent variable adjusting for smoking status, alcohol consumption status and other SNPs in the locus. All reported *p*-values are two-sided and statistical analyses were performed using the R statistical computing platform (version 3.4.2).

## Results

### Study Sample Characteristics

Characteristics of cases and controls by ancestry group are shown in Table 1. The mean age of diagnosis was similar in the Black and Mixed Ancestry samples (59.6 and 60.7 years, respectively). The male to female ratio was 0.98 in Black cases and 1.92 in Mixed Ancestry cases. In addition, Mixed Ancestry cases had higher rates of ever-smokers (94%) and alcohol drinkers (81%) compared with Black cases (61 and 62%, respectively).

### Single Variant Association Analysis

The results for the case-control analysis in the two South African populations are summarized in Table 2. Three of the five 2q33 variants tested were marginally associated with a higher risk of ESCC in the Mixed Ancestry population. These were *CASP8* rs10931936 (OR = 1.39, 95% CI = 1.02–1.90, *p* = 3.52 × 10^−2^), *TRAK2* rs7578456 (OR = 1.58, 95% CI = 1.22–2.05, *p* = 2.59 × 10^−4^), and *TRAK2* rs2244438 (OR = 1.55, 95% CI = 1.16–2.07, *p* = 2.30 × 10^−3^). Of these, rs7578456 and rs2244438 remained significant after Bonferroni multiple testing correction (*P* < 0.005). A suggestive association was also noted for *CASP8* rs3769823 (OR = 1.26, 95% CI = 0.97–1.63, *p* = 0.076). Interestingly, none of these variants were associated with ESCC in the Black South African population. None of the SNPs on 6p21 tested positive for association with ESCC in either the Black or the Mixed Ancestry populations.

**Table 2:** Case-control association results for Black and Mixed Ancestry South Africans.

					Black (463 cases, 480 controls)			Mixed Ancestry (269 cases, 288 controls)		
SNP	Base change^a^	Locus	Position^b^	Gene^c^	MAF (ca/co)^d^	OR (95% CI)	*P*-value^e^	MAF (ca/co)	OR (95% CI)	*P*-value
rs3769823	G > A	2q33	202122995	*CASP8*	0.35/0.35	0.98 (0.80–1.20)	0.841	0.38/0.33	1.26 (0.97–1.63)	0.076
rs10931936	C > T	2q33	202143928	*CASP8*	0.18/0.20	0.89 (0.70–1.13)	0.354	0.23/0.18	1.39 (1.02–1.90)	0.035
rs13016963	G > A	2q33	202162811	*ALS2CR12*	0.33/0.34	0.96 (0.79–1.17)	0.68	0.33/0.29	1.18 (0.91–1.54)	0.209
rs7578456	G > A	2q33	202235348	*TRAK2/ALS2CR12*	0.42/0.41	1.04 (0.85–1.26)	0.718	0.43/0.32	1.58 (1.22–2.05)	2.59E-04
rs2244438	G > A	2q33	202252539	*TRAK2*	0.27/0.28	0.97 (0.78–1.20)	0.777	0.28/0.20	1.55 (1.16–2.07)	2.30E-03
rs911178	C > T	6p21	28574415	*ZBED9*	0.17/0.18	0.99 (0.77–1.27)	0.92	0.12/0.10	1.14 (0.77–1.69)	0.502
rs3763338	G > A	6p21	28894311	*TRIM27*	0.24/0.26	0.87 (0.69–1.08)	0.212	0.18/0.20	0.89 (0.64–1.22)	0.462
rs2844695	T > C	6p21	30936014	*MUC21/HCG21*	0.23/0.21	1.05 (0.83–1.32)	0.542	0.28/0.27	1.11 (0.69–1.56)	0.248
rs17533090	G > T	6p21	32590722	*HLA-DQA1*	0.14/0.14	0.97 (0.73–1.28)	0.841	0.15/0.13	1.14 (0.80–1.64)	0.462
rs1536501	C > T	6p21	33727885	*LEMD2/IPSK3*	0.26/0.24	1.14 (0.92–1.42)	0.233	0.16/0.15	1.10 (0.78–1.55)	0.566

*^a^Major allele > minor allele. ^b^Position based on the human genome build 37 (GRCh37). ^c^Gene location or nearest gene. ^d^Minor allele frequency in cases and controls. ^e^p-values from Pearson’s chi-squared test.*

### Gene-Environment Interaction Analysis

An analysis of alcohol consumption and cigarette smoking showed that there was no significant difference in association with rs10931936, rs7578456, and rs2244438 in the Mixed Ancestry sample across strata for tobacco smoking or alcohol consumption status, except for rs7578456 that was statistically significantly associated with risk in alcohol drinkers (OR = 1.61; 95% CI = 1.18–2.20, *p* = 0.003) but not in non-alcohol drinkers (OR = 1.55, 95% CI = 0.94–2.55, *p* = 0.085) (Table 3). As a further evidence for the lack of gene-environment interactions, the effect sizes in smokers and alcohol-drinkers were not substantially higher than those observed in all cases combined, while the number of individuals never smokers and non-drinker categories were too low to provide informative risk estimates (see Table 1).

**Table 3:** Case-control association results by tobacco smoking and alcohol consumption status in Mixed Ancestry South Africans^a^.

	Ever-smokers^b^			Never-smokers		
SNP	MAF (ca/co)^c^	OR (95% CI)	*P*-value^d^	MAF (ca/co)	OR (95% CI)	*P*-value
rs10931936	0.24/0.19	1.36 (0.98–1.89)	0.067	0.25/0.12	2.38 (0.75–7.49)	0.13
rs7578456	0.44/0.34	1.47 (1.11–1.94)	0.006	0.46/0.26	2.44 (1.05–5.68)	0.035
rs2244438	0.28/0.21	1.52 (1.12–2.07)	0.007	0.35/0.16	2.75 (1.08–7.04)	0.03

	**Alcohol drinkers^e^**			**Non-alcohol drinkers**		
**SNP**	**MAF (ca/co)**	**OR (95% CI)**	***P*-value**	**MAF (ca/co)**	**OR (95% CI)**	***P*-value**

rs10931936	0.23/0.19	1.28 (0.87–1.87)	0.206	0.26/0.17	1.68 (0.93–3.05)	0.083
rs7578456	0.42/0.31	1.61 (1.18–2.20)	0.003	0.44/0.34	1.55 (0.94–2.55)	0.085
rs2244438	0.26/0.19	1.45 (1.02–2.07)	0.037	0.34/0.20	2.06 (1.19–3.53)	0.008

*^a^Only SNPs associated with ESCC risk in the overall case-control analysis are shown. ^b^Subjects who had smoked at some point in their life. ^c^Minor allele frequency in cases and controls. ^d^p-values from Pearson’s chi-squared test. ^e^Subjects who consumed any alcoholic beverage at least once every week.*

### Linkage Disequilibrium and Haplotype Analysis

**FIGURE 1:**
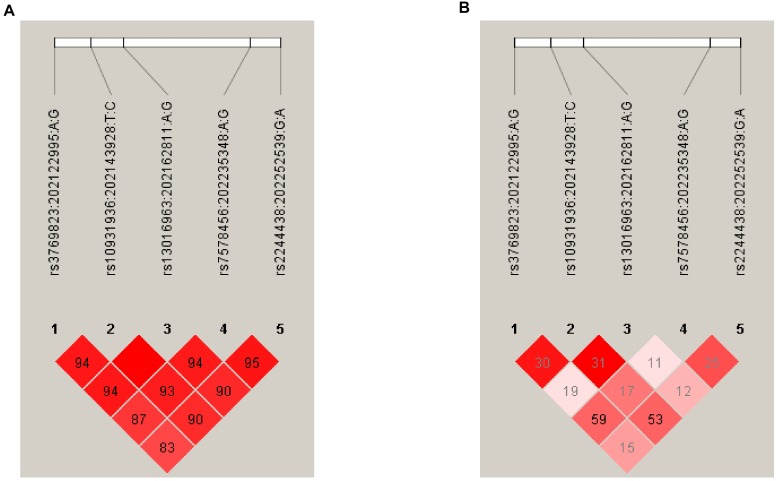
Linkage disequilibrium plots of the five SNPs within the 2q33 locus in Africans **(A)** and Asians **(B)**. Each panel shows the LD plot of the 2q33 locus bounded by rs3769823 and rs2244438 (chr2: 202122995–202252539). Correlation coefficients (*r*^2^ and D’) were inferred using Haploview (Barrett et al., 2005). Pairwise correlations were based on the African (AFR) and Asian (EAS) Ancestry panels from the 1000 Genomes Project Phase 3. Color intensity of squares (white to red) indicates the strength of LD (low to high) by D’ values, while numbers within squares refer to *r*^2^-values for pairwise correlation. D’ and *r*^2^ refer to different statistical methods to measure LD between alleles; *r*^2^ is preferred to predict one allele given the other, whereas D’ is mainly used to assess recombination patterns such as haplotype blocks.

Correlation coefficients (D^*I*^ and *r*^2^) and linkage disequilibrium (LD) plots for SNPs at 2q33 were computed using the African and Asian population samples from the 1000 Genomes Project (see Materials and Methods) allowing a comparison of the LD structure between these ethnic groups. This shows that Africans have a lower level of LD (*r*^2^ range: 0.15–0.59) across the 2q33 locus compared with Asians (*r*^2^ range: 0.51–0.96) (Figure 1). Notably, there was a moderate correlation between rs3769823 and rs7578456 (*r*^2^ = 0.59) and between rs10931936 and rs2244438 (*r*^2^ = 0.53) in Africans, suggesting that the association of these variants with ESCC risk in the current study may be driven by one or two independent association signals. No considerable correlation was observed for SNPs at the 6p21 locus (*r*^2^≤ 0.01; data not shown).

In the Mixed Ancestry sample, a marginal association was observed for the haplotype consisting of all minor alleles at 2q33 (ATAAA; OR = 1.54, 95% CI = 1.06–2.25, *p* = 0.024) compared with carriers of all common alleles (GCGGG; Supplementary Table 1). However, no increased risk was achieved by co-occurrence of the five minor alleles on the same haplotype compared with the risk predicted in the single variant test. Haplotype analysis of the five SNPs at 6p21 showed no evidence of association with ESCC in both the Black and Mixed Ancestry samples (*p* ≥ 0.09; Supplementary Table 2).

### Logistic Regression Analysis

The multivariate logistic regression analysis revealed marginal associations with rs2244438 in the Mixed Ancestry sample after we controlled for the effect of the other SNPs in the region (rs3769823, rs10931936, rs13016963, rs7578456), although none of these associations were statistically significant after Bonferroni correction (highest OR = 1.19, smallest *p* = 0.008) (Table 4). Conversely, rs7578456 and rs10931936 were no longer significantly associated with risk after adjusting for rs2244438 (*p* = 0.399 and 0.354, respectively), indicating that rs2244438 was the variant driving the association at 2p33.

**Table 4:** Logistic regression analysis for association of 2q33 SNPs with ESCC risk in Mixed Ancestry South Africans.

	rs3769823		rs10931936		rs13016963		rs7578456		rs2244438	
SNP	OR (95% CI)^a^	*P*-value^b^	OR (95% CI)	*P*-value	OR (95% CI)	*P*-value	OR (95% CI)	*P*-value	OR (95% CI)	*P*-value
rs3769823	NA	NA	0.96 (0.92–1.02)	0.171	0.97 (0.93–1.01)	0.13	0.98 (0.94–1.02)	0.283	0.97 (0.93–1.01)	0.154
rs10931936	0.99 (0.94–1.05)	0.792	NA	NA	0.96 (0.92–1.01)	0.147	0.98 (0.93–1.02)	0.336	0.98 (0.93–1.02)	0.354
rs13016963	1.03 (0.97–1.10)	0.343	1.02 (0.95–1.09)	0.482	NA	NA	1.02 (0.96–1.09)	0.47	1.02 (0.95–1.09)	0.583
rs7578456	1.05 (0.99–1.11)	0.081	1.06 (1.00–1.13)	0.032	1.06 (1.00–1.13)	0.037	NA	NA	1.03 (0.97–1.09)	0.399
rs2244438	1.19 (1.04–1.35)	0.015	1.15 (1.01–1.32)	0.038	1.19 (1.05–1.37)	0.008	1.18 (1.02–1.37)	0.024	NA	NA

*^a^Estimates for each SNP adjusting for smoking status, alcohol consumption status and each other SNP in the locus. ^b^p-value from Wald test.*

## Discussion

This study investigated the association between common genetic polymorphisms at 2q33 and 6p21 and the risk of ESCC in two South African populations. These variants were previously reported to be associated with higher risk for both ESCC and other cancer types in the Chinese population (Shen et al., 2014; Zhang et al., 2017; Zhao et al., 2017). Our analysis revealed three SNPs at 2q33 (rs10931936, rs7578456, rs2244438) that conferred an increased risk of ESCC in the Mixed Ancestry population, thus replicating the association signals identified in the Chinese study (Zhao et al., 2017). A similar pattern of association was also noted for rs3769823, but without statistical significance. Of these, rs2244438 that maps to a genomic region harboring *TRAK2* was independently associated with ESCC risk after adjusting for the other SNPs at 2q33. These findings point to a single causal variant at 2q33 and suggest that multiple association signals at this locus may result from the high correlation with the causal variant. We should also note that rs2244438, unlike rs7578456 and rs10931936, lies within an exon of *TRAK2* and therefore is more likely to have a functional effect on the encoded protein.

All associations observed in the Mixed Ancestry population were also detected by strata of smoking and alcohol exposure. In addition, these associations were not strengthened when the analysis was restricted to ever-smokers or alcohol drinkers relative to the full sample. Our findings are suggestive of no gene-environment interactions at the 2q33 locus, which is in line with the lack of interaction with smoking for Chinese lung cancer risk (Zhao et al., 2017). However, the proportion of never-smokers and non-drinkers in the Mixed Ancestry sample was very small, with low power to detect associations in these subgroups. Whether these two polymorphisms increase the risk of ESCC upon exposure to tobacco smoke or alcohol requires confirmation by analysis of a larger sample.

This is the first study to report that SNPs mapping to *TRAK2* were significantly associated with ESCC risk in African populations and support previous findings of an association between rs2244438 and ESCC and lung cancer risk in the Chinese (Zhao et al., 2017). The trafficking kinesin-binding protein 2 (*TRAK2*, also known as *GRIF-1*) is a member of a coiled-coil family of proteins with a role in regulating protein and organelle transport in cells (Brickley and Stephenson, 2011). Downregulation of this kinesin-associated protein may therefore cause dysfunctional cell signaling and potentially result in carcinogenesis. The variant rs2244438 is located within an exon with evolutionary constraints, and the G to A transition at this site results in the nonsynonymous change from threonine (Thr) to isoleucine (Ile) at the residue position of 528 on *TRAK2*. Functional effect prediction programs such as SIFT^2^ and PolyPhen^3^ suggested a damaging effect associated with this genetic polymorphism. Secondary structure prediction indicates that residue 528 is located at a disordered region of molecular surface, and that the Thr to Ile change probably disturbs the adjacent secondary structure. The *TRAK2* gene at 2q33 is therefore a credible candidate for containing the causal variant driving the association at this locus, and the effect of rs2244438 on gene expression will require functional follow-up to determine its potential pathogenic effect on the protein.

We also performed the first analysis of the MHC region in relation to ESCC risk in the two South African populations described in this paper. The MHC encodes a set of cell surface glycoproteins known as human leukocyte antigens (HLA), that are critical for innate and adaptive immune response in humans (Horton et al., 2004). Loss of heterozygosity and DNA hypermethylation in the MHC region resulting in the downregulation of HLA class I and class II genes are common and well-recognized event in esophageal tumors (Nie et al., 2001; Yang et al., 2008; Zhao et al., 2011). Germline variants at this locus have been shown to confer higher risk of ESCC in Chinese populations (Shen et al., 2014; Zhang et al., 2017). However, we could not replicate such association with these variants in South African populations, although they were observed at a high enough frequency (≥10%) to detect suggestive associations with ESCC.

Our study suggests that genetic risk variants at the 2q33 locus are shared between the Chinese populations and the Mixed Ancestry population of South Africa. The Mixed Ancestry population from the Western Cape is an admixed population that originated from the union of different ethnic groups, receiving 9–11% of the ancestral contribution from Asians (de Wit et al., 2010). Thus, it is possible that genetic risk markers commonly found in the Chinese could have been inherited in the Mixed Ancestry population of South Africa. The relatively small and variable Asian genetic component across Mixed Ancestry individuals may also explain the weaker genetic associations with ESCC commonly observed in this ethnic group compared with the Chinese (Zhao et al., 2017). Population stratification could have contributed to the associations observed in this ethnic group, but this could only be resolved by high throughput genotyping of very large numbers of SNPs with appropriate statistical correction for any differences observed (Teo et al., 2010). No evidence of association was observed in the Black population, which is consistent with our previous studies that failed to detect several associations reported in Chinese GWAS (Bye et al., 2011, 2012; Chen et al., 2019). The Black South African samples in this study are derived almost entirely from the Xhosa-speaking population, which is a genetically conserved population that received little or no ancestral contribution from other ethnic groups across generations. It is well established that African genomes have greater genetic diversity and lower LD map compared with Asian and European genomes (Campbell and Tishkoff, 2008). As an example, the five SNPs at 2q33 tested in this study were in lower LD in Africans (max *r*^2^ = 0.59) compared with the Chinese (max *r*^2^ = 0.96), and the number of private haplotypes was higher in Black individuals as compared with Mixed Ancestry individuals. The genetic risk variants reported in the Chinese population may therefore not capture the actual causal variants in the Black African population. There is also a possibility that the causal variants may have arisen after the migration of humans out of Africa and are therefore not present in Black African populations. Finally, differences in genetic associations between Asian and African populations may in part reflect variability in environmental exposures between ethnic groups, or technical issues such as small sample sizes which are not well powered to detect modest genetic effects. Fine-mapping of ESCC susceptibility loci should be carried out to provide additional biological insights and identify the causal variants driving the association in African populations.

## Conclusion

In conclusion, our study reports a possible association between a gene involved in cellular trafficking and ESCC in the Mixed Ancestry population from the Western Cape province of South Africa, as previously described in a Chinese population. If validated in larger independent studies, these variants may aid in the identification of individuals at high risk of developing ESCC, who would benefit from early screening and prevention strategies. These variants may also represent novel targets for functional follow-up aimed at elucidating the underlying biological mechanisms of esophageal carcinogenesis and identifying targeted therapies tailored toward ESCC patients with specific genetic markers. We did not detect associations with genetic variants at 6p21 that were previously reported in Chinese populations, thus providing further support for the lack of replication of genetic findings across ethnic groups that may reflect differences in genetic architecture and environmental exposure. Therefore, GWAS and fine-mapping studies in African populations are required to increase our understanding of the genetic contribution to ESCC and to gain further insights into the genetic heterogeneity of the disease. In addition, the combined effects of genes and common environmental factors may play a role in interpreting association data across populations and should always be considered in the study of complex diseases such as esophageal cancer.

## Ethics Statement

Ethical approval for the study was obtained from the joint University of Cape Town/Groote Schurz Hospital Research Ethics Committee and the University of Stellenbosch/Tygerberg Hospital Ethics Committee. All subjects gave written informed consent in accordance with the Declaration of Helsinki.

## Author Contributions

MM preformed the laboratory work, contributed to the statistical analysis, and wrote the first draft of the manuscript. CM revised the manuscript critically for important intellectual content. MP conceived and designed the study. All authors contributed to and read the final version of the manuscript revision.

## Conflict of Interest Statement

The authors declare that the research was conducted in the absence of any commercial or financial relationships that could be construed as a potential conflict of interest. The handling Editor declared a shared affiliation, though no other collaboration, with one of the authors CM at the time of review.

## Footnotes

1 www.1000genomes.org

2 http://sift.jcvi.org

3 http://genetics.bwh.harvard.edu/pph
