# Supplementary material for: The Relationship Between Environmental Exposure and Genetic Architecture of the 2q33 Locus With Esophageal Cancer in South Africa
Source: Front Genet. 2019 May 1;10:406. doi: 10.3389/fgene.2019.00406 (PMC6504765; doi:10.3389/fgene.2019.00406)
Supplement: Supplementary file 1 [file Table_1.DOCX]

| **Supplementary Table 1. Haplotype association results for SNPs at 2q33 in Black and Mixed Ancestry South Africans**^1^ | | | | | | | |  |
| --- | --- | --- | --- | --- | --- | --- | --- | --- |
| Black | | | |  | Mixed Ancestry | | | |
| Haplotype^2^ | MAF (ca/co)^2^ | OR (95% CI) | P-value^3^ |  | Haplotype | MAF (ca/co) | OR (95% CI) | P-value |
| G - C - G - G - G | 0.42/0.45 | Ref |  |  | G - C - G - G - G | 0.46/0.50 | Ref |  |
| **A** - **T** - **A** - **A** - **A** | 0.16/0.17 | 1.05 (0.79-1.41) | 0.74 |  | **A** - **T** - **A** - **A** - **A** | 0.19/0.14 | 1.54 (1.06-2.25) | 0.024 |
| G - C - **A** - G - G | 0.08/0.08 | 1.17 (0.79-1.72) | 0.417 |  | **A** - C - G - G - G | 0.06/0.08 | 0.81 (0.43-1.21) | 0.136 |
| **A** - C - G - **A** - G | 0.07/0.08 | 0.96 (0.65-1.43) | 0.862 |  | G - C - **A** - G - G | 0.04/0.07 | 0.74 (0.41-1.35) | 0.325 |
| **A** - C - **A** - **A** - G | 0.07/0.06 | 1.24 (0.81-1.89) | 0.322 |  | **A** - C - G - **A** - G | 0.06/0.05 | 1.49 (0.83-2.71) | 0.177 |
| G - C - G - **A** - **A** | 0.06/0.06 | 0.95 (0.61-1.48) | 0.823 |  |  |  |  |  |
| G - C - G - **A** - G | 0.06/0.05 | 1.29 (0.81-2.05) | 0.279 |  |  |  |  |  |
| SNP order: rs3769823 - rs10931936 - rs13016963 - rs7578456 - rs2244438 | | | | | |  |  |  |
| ^1^Only haplotypes with an estimated frequency in controls ≥5% were tested | | | | | |  |  |  |
| ^2^Letters correspond to the nucleotide of each SNP in the corresponding order; haplotypes ordered by frequency in controls; risk alleles highlighted in bold | | | | | | | | |
| ^3^Estimated effect of each haplotype relative to the reference haplotype with non-risk alleles | | | | | | | |  |

| **Supplementary Table 2. Haplotype association results for SNPs at 6p21 in Black and Mixed Ancestry South Africans**^1^ | | | | | | | |  |
| --- | --- | --- | --- | --- | --- | --- | --- | --- |
| Black | | | |  | Mixed Ancestry | | | |
| Haplotype^2^ | MAF (ca/co)^2^ | OR (95% CI) | P-value^3^ |  | Haplotype | MAF (ca/co) | OR (95% CI) | P-value |
| C - G - T - G - C | 0.38/0.38 | Ref |  |  | C - G - T - G - C | 0.50/0.53 | Ref |  |
| C - **A** - T - G - C | 0.13/0.15 | 0.89 (0.65-1.20) | 0.439 |  | C - **A** - T - G - C | 0.13/0.13 | 1.02 (0.68-1.52) | 0.92 |
| C - G - T - G - **T** | 0.15/0.12 | 1.24 (0.91-1.69) | 0.168 |  | C - G - T - **T** - C | 0.11/0.09 | 1.27 (0.81-1.98) | 0.294 |
| **T** - G - T - G - C | 0.08/0.10 | 0.77 (0.53-1.12) | 0.174 |  | C - G - T - G - **T** | 0.11/0.08 | 1.48 (0.94-2.34) | 0.09 |
| C - G - **C** - G - C | 0.11/0.08 | 1.29 (0.93-1.52) | 0.201 |  | **T** - G - T - G - C | 0.07/0.07 | 1.12 (0.66-1.89) | 0.671 |
| C - G - T - **T** - C | 0.08/0.07 | 1.13 (0.76-1.69) | 0.532 |  | C - G - **C** - G - C | 0.06/0.05 | 1.12 (0.77-1.68) | 0.402 |
| C - **A** - T - G - **T** | 0.04/0.05 | 0.87 (0.54-1.41) | 0.572 |  |  |  |  |  |
| SNP order: rs911178 - rs3763338 - rs2844695 - rs17533090 - rs1536501 | | | | | |  |  |  |
| ^1^Only haplotypes with an estimated frequency in controls ≥5% were tested | | | | | |  |  |  |
| ^2^Letters correspond to the nucleotide of each SNP in the corresponding order; haplotypes ordered by frequency in controls; risk alleles highlighted in bold | | | | | | | | |
| ^3^Estimated effect of each haplotype relative to the reference haplotype with non-risk alleles | | | | | | | |  |
